# The fractionalization of physical activity throughout the week is associated with the cardiometabolic health of children and youth

**DOI:** 10.1186/1471-2458-13-554

**Published:** 2013-06-06

**Authors:** Ian Janssen, Suzy L Wong, Rachel Colley, Mark S Tremblay

**Affiliations:** 1 Department of Public Health Sciences, School of Kinesiology and Health Studies, Queen’s University, Kingston, ON, Canada; 2 Health Analysis Division, Statistics Canada, Ottawa, ON, Canada; 3 Healthy Active Living and Obesity Research Group, Children’s Hospital of Eastern Ontario Research Institute, Ottawa, ON, Canada

## Abstract

**Background:**

The influence of the fractionalization of moderate- to vigorous- intensity physical activity (MVPA) throughout the week on the health of children is unknown. We compared cardiometabolic risk factors in physically active children who accumulated their weekly MVPA in different patterns.

**Methods:**

We studied 745 participants aged 6–19 years. MVPA was measured using accelerometers over 7 days. Three groups were created: *Insufficiently Active,* <60 minutes/day of MVPA on average; *Infrequently Active,* ≥60 minutes/day of MVPA on average but exceeding the 60 minute target <5 days; and *Frequently Active,* ≥60 minutes/day MVPA on average and exceeding the 60 minute target ≥5 days. Percentile scores for 8 cardiometabolic risk factors were determined.

**Results:**

The least favorable cardiometabolic risk factor profile was observed in the *Insufficiently Active* group. The *Frequently Active* group had more favorable (5–6 percentile unit difference) diastolic blood pressure, HDL-cholesterol, insulin resistance, and metabolic syndrome scores than the *Infrequently Active* group, although only the difference for insulin resistance was statistically significant (P < 0.05). These differences remained after controlling for the weekly volume of MVPA.

**Conclusion:**

The fractionalization of MVPA throughout the week was associated with insulin resistance.

## Background

Physical activity guidelines of the World Health Organization [1], United States [2,3], and Canada [4] all recommend that school-aged children and youth accumulate at least 60 minutes of moderate- to vigorous-intensity physical activity (MVPA) *every day* for health benefits. A recent systematic review on the health benefits of physical activity in school-aged children and youth noted that, while there is evidence to conclude that an average of 60 minutes/day of MVPA is associated with meaningful health benefits, there is no evidence to support the daily frequency recommendation [5]. In other words, the fractionalization of MVPA throughout the week may not be important, and it is possible that an *average* of 60 minutes/day of MVPA is just as beneficial as 60 minutes *every day*. Consistent with this observation, physical activity guidelines for adults have changed from the recommendation to accumulate 30 minutes of MVPA on most or all days of the week [6,7] to the recommendation to accumulate 150 minutes over the week [1,3,4]. This change was made because existing evidence does not permit researchers to say, for example, whether the benefits of 30 minutes of MVPA 5 days/week are different from the benefits of 50 minutes of MVPA on 3 days/week [8,9].

It is unclear as to whether the fractionalization of a young person’s volume of MVPA throughout the week impacts their health. Only a few studies have compared the benefits of several week days of MVPA to those of a few days, when the dose of MVPA is controlled for, and these studies are limited to adults. One of these studies consisted of a prospective cohort of older men followed for mortality over 9 years [10]. Within that study physically active men (≥1000 kcal/week) were classified as infrequently (active 1–2 days/week) or regularly (active 3–7 days/week) active based upon the number of days they exercised each week. Compared to inactive men, mortality risk was reduced by 15% in infrequently active men and by 36% in regularly active men. The twofold greater risk reduction in regularly active than infrequently active men suggests that the recommendation to fractionalize weekly MVPA over several days may be relevant. Additional studies, particularly in young people, are needed.

Our objective was to compare cardiometabolic risk factors in physically active children and youth (i.e., ≥60minutes/day on average) who accumulate their weekly MVPA in different patterns (i.e., ≥60 minutes on 1–4 days/week vs. 5–7 days/week). We hypothesized that, after controlling for the total dose of MVPA, children and youth who were active more frequently would have more favorable cardiometabolic risk factors than children and youth who are active less frequently. This hypothesis was based on the knowledge that, in addition to chronic effects, MVPA has acute effects on several cardiometabolic risk factors that can last for as long as three or four days [11].

## Methods

### Data source

Data are from the Canadian Health Measures Survey (CHMS), a nationally representative sample of Canadians aged 6 to 79 years living in private households [12-15]. Residents of Indian Reserves or Crown lands, institutions, certain remote regions, and full-time members of the Canadian Forces are excluded. Approximately 96% of Canadians are represented. The CHMS involved two components: 1) an in-home interview (completed by proxy for those aged <12 years) to gather information on socio-demographic characteristics, health behaviors, and environmental factors, and 2) a mobile examination clinic visit for direct physical measures and biospecimen collection. To be included in this study, participants had to be 6–19 years old, have acceptable accelerometry measures of MVPA, and have measures available for all of the cardiometabolic risk factors.

Ethics approval was obtained from Health Canada’s Research Ethics Board [15]. Informed written consent was obtained from respondents aged 14 years or older. For a child, a parent/guardian provided written consent, in addition to written assent from the child. Data were collected at 15 sites from March 2007 through February 2009.

Of the households selected to participate in the CHMS, the response rate was 69.6%. Within each household, one (if there were no children aged 6–11 years) or two (if there was ≥ one child aged 6–11 years) members were randomly chosen to participate. Half of the CHMS participants made up the fasted subsample (these participants completed their clinic visit in the morning) that was examined in this paper. Of the selected 6–19 year olds, 86.8% completed the household questionnaire, 87.5% of those participated in the mobile clinic visit, and 85.4% of those were fasted for a total of 933. The overall response rate for the fasted 6–19 year olds is therefore 45.2%. An additional 188 were excluded because of missing or incomplete data, leaving a final sample size of 745.

### Measurement of physical activity

After the clinic visit, participants were asked to wear an Actical accelerometer (Phillips – Respironics, Oregon, USA) during their waking hours over their right hip on an elasticized belt for 7 consecutive days. The monitors started collecting data at midnight following the clinic visit. The Actical measures time-stamped accelerations in all directions and provides an indication of physical activity intensity. The digitized values were summed over 1 minute intervals, resulting in 10,080 count per minute (cpm) values over the 7 day measurement period. The Actical has been validated to measure physical activity in children and youth [16,17].

#### Cleaning and reduction of accelerometer data

Accelerometer data quality were assessed to determine whether files should be included in analyses [18]. Published guidelines were followed to identify and remove days with incomplete (invalid) accelerometer wear time [18,19]. A valid day was defined as ≥10 hours of wear time, which was determined by subtracting nonwear time from 24 hours. Nonwear time was defined as a period of ≥60 consecutive minutes of zero counts, with allowance for 1 to 2 minutes of counts between 0 and 100.

The next data cleaning step involved removing participants with an insufficient number of days of valid wear time. Only participants with ≥4 valid days were included [18]. A 4–5 day accelerometer monitoring period for MVPA has a test-retest reliability of 0.7 to 0.8 among children and youth [20]. Of those child and youth CHMS participants who wore the accelerometer, 84.8% had at least 4 valid days (8.2% with 4 days, 12.7% with 5 days, 24.0% with 6 days, 39.8% with 7 days) [21].

#### Derivation of MVPA variables

Time spent in MVPA was determined for each day with valid wear time by summing minutes with values ≥1500 cpm [22]. Average daily minutes of MVPA was calculated for each participant. The number of days over the 7 day measurement period in which the participants accumulated ≥60 minutes of MVPA was also determined.

Physical activity recommendations are that school-aged children and youth accumulate ≥60 minutes of MVPA *daily*. Participants in this study were divided into the following 3 groups based on whether they achieved the 60 minute/day target on average over the 7 day measurement period, and if so, the number of days that this target was achieved: 1) *Insufficiently Active* = <60 minutes/day of MVPA on average over the 7 day measurement period (N = 455); 2) *Infrequently Active* = ≥60 minutes/day of MVPA on average over the 7 day measurement period, and active for ≥60 minutes on 1 to 4 days over the 7 days (N = 106); and 3) *Frequently Active* = ≥60 minutes/day MVPA on average over the 7 day measurement period, and active for ≥60 minutes on 5 to 7 days over the 7 days (N = 184). The *Infrequently Active* and *Frequently Active* groups were differentiated using the 5 day cut-point because this cut-point resulted in the most comparably sized groups.

To maximize the sample size, participants with less than 7 valid days of wear time were classified into the 3 MVPA groups based on an equivalent percentage of active days for the number of days they had valid wear time. For participants with 7 out of 7 days of valid wear time, 4 out of 7 active days equates to having 57% of all valid days as active days, for participants with 6 days of valid wear time this is roughly equivalent to 3 active days (50%), for participants with 5 days of valid wear time this is roughly equivalent to 3 active days (60%), and for participants with 4 days of valid wear time, this is roughly equivalent to 2 active days (50%).

### Measurement of cardiometabolic risk factors

Cardiometabolic risk factor values vary by sex and change with growth and maturation [23-25]. Therefore, percentile scores by sex and age were computed for each cardiometabolic risk factor, and these percentile scores were used in all regression analyses. Thus, participants with percentile scores of 2%, 16%, 50%, 84%, and 98% would reflect individuals with risk factor values for their sex and age that were 2 SD below the mean, 1 SD below the mean, at the mean, 1 SD above the mean, and 2SD above the mean, respectively.

#### Obesity

Height was measured to the nearest 0.1 cm using a ProScale M150 digital stadiometer (Accurate Technology Inc., Fletcher, USA) and weight was measured to the nearest 0.1 kg with a Mettler Toledo VLC scale (Mettler Toledo Canada, Mississauga, Canada). The body mass index (BMI) was calculated as weight divided by height squared (kg/m^2^). Waist circumference was measured with an anthropometric tape at the end of a normal expiration to the nearest 0.1 cm at the mid-point between the last rib and iliac crest [26]. Overweight and obesity were determined using the International Obesity Task Force BMI thresholds [23].

#### Blood pressure

Blood pressure was measured with the BpTRU™ BP-300 (BpTRU Medical Devices Ltd., Coquitlam, British Columbia) [27]. The device automatically inflates and deflates the arm cuff and uses an oscillometric technique to calculate systolic and diastolic blood pressure. It has passed international validation protocols [12,28]. Once appropriate seat height, arm positioning, and cuff size were determined, a five minute quiet rest was followed by a minimum of six measurements taken one minute apart. Systolic and diastolic blood pressure values were calculated by averaging the last 5 of 6 measures.

#### Blood measures

Fasting blood samples were taken and analyzed at the Health Canada Laboratory (Bureau of Nutritional Sciences, Nutrition Research Division). The lipid/lipoprotein measures considered here were high density lipoprotein (HDL)-cholesterol and triglycerides. We did not examine low density lipoprotein-cholesterol because it is poorly correlated with MVPA in children and youth [29]. HDL-cholesterol and triglycerides were measured using a non-HDL precipitation method and enzymatic method, respectively, and were performed on the Vitros 5,1FS (Ortho Clinical Diagnostics). Insulin resistance was estimated according to the homoeostasis model assessment (HOMA) as the product of fasting glucose (mmol/L) and insulin (μU/mL) divided by 22.5 [30]. Insulin was measured using a solid-phase, two-site chemiluminescent immunometric assay (Immulite 2000 by DPC). Glucose was measured on the Vitros 5,1FS (Ortho Clinical Diagnostics).

#### Metabolic syndrome

Percentile scores of the seven individual risk factors were averaged for each participant to create a clustered metabolic syndrome risk score [31]. HDL-cholesterol scores were multiplied by −1 prior to incorporating them into the metabolic syndrome score.

### Covariates

Covariates included visible minority status (white or other) and total household income adjusted for the number of people in the household (lowest quartile vs. other). Age and sex were not included as covariates because the cardiometabolic risk factor percentile scores were age- and sex-specific.

### Statistical analyses

Statistical analyses were performed using SAS v9.1 and SUDAAN v10. Some of the cardiometabolic risk factor values were positively skewed (triglycerides and HOMA) and were log transformed prior to analyses. All analyses were weighted to obtain estimates that are representative of the Canadian population [32]. To account for survey design effects, 95% confidence intervals (CI) were estimated using the bootstrap technique [33,34], with the degrees of freedom specified as 11 [32].

Mean and prevalence estimates were produced. Linear regression tests were used to assess the univariate relations between MVPA and the cardiometabolic risk factors. Differences between cardiometabolic risk factors across the three physical activity groups were assessed using general linear models. The 95% CI of the parameter estimates (b) from these regression models were used to identify group differences. Visible minority status and household income were included as covariates in the models. Two models were run for each cardiometabolic risk factor. The first model included the 3 physical activity groups and the covariates. The second model included the 3 physical activity groups, the covariates, and the volume of MVPA (average min/day). There was a 21 minute/day difference in the average daily MVPA in the *Infrequently Active* and *Frequently Active* groups, and the second model controlled for the volume of MVPA to estimate the independent effects of the weekly frequency of being active for ≥60 minutes.

Statistical results were interpreted as recommended by the British Medical Journal Group [35]. These recommendations indicate that: 1) CI for effect sizes are preferable to p-values, 2) inferences drawn by comparing p-values are dubious, and 3) interpretation of a lack of significance as evidence of no effect is usually incorrect.

Based on a sample size of 106 or more per group, a p value of 0.05, and assuming a standard deviation for the cardiometabolic risk factor percentile scores of 15 within each group, the study had an 80% power to detect a 5 unit difference between the mean percentile scores of two groups.

## Results

### Descriptive characteristics

A description of the 745 participants is in Table 1. The average age was 12.4 years, 48.7% were male, and 25.6% were overweight or obese. The average daily MVPA was 55.1 minutes. A total of 455 participants (61.1%) accumulated <60 minutes/day of MVPA on average (*Insufficiently Active* group), 106 (14.2%) accumulated ≥60 minutes/day of MVPA on average but were only active for ≥60 minutes on 1–4 days of the week (*Infrequently Active* group), and 184 (24.7%) accumulated ≥60 minutes/day of MVPA on average and were active for ≥60 minutes on 5–7 days of the week (*Frequently Active* group). The average daily minutes of MVPA in these 3 groups were 36.1 (95% CI: 34.1, 38.2), 70.0 (95% CI: 67.7, 72.3), and 90.9 (95% CI: 85.0, 96.8), respectively.

**Table 1:** **Descriptive characteristics**: **6–19 year old 2007/09 Canadian health measures survey participants**

**Variable**	**Total sample**	***Insufficiently active***	***Infrequently active***	***Frequently active***
Age, years	12.4 (12.1, 12.8)	13.1 (12.6, 13.6)	12.0 (10.8, 13.2)	11.1 (10.3, 12.0)
MVPA				
Average min/day (mean)	55.1 (49.5, 60.7)	36.1 (34.1, 38.2)	70.0 (67.7, 72.3)	90.9 (85.0, 96.8)
≥60 min/day on average (%)	39.9 (31.8, 48.6)	0	100	100
BMI, kg/m^2^	20.3 (19.7, 20.8)	21.0 (20.2, 21.8)	19.4 (18.3, 20.6)	18.9 (17.9, 19.8)
Waist circumference, cm	68.7 (67.2, 70.2)	70.7 (68.3, 73.0)	66.1 (63.0, 69.2)	66.5 (62.3, 68.6)
Systolic blood pressure, mmHg	96.3 (95.1, 97.5)	97.1 (95.5, 98.7)	95.9 (93.7, 98.1)	94.7 (93.5, 95.9)
Diastolic blood pressure, mmHg	62.2 (61.1, 63.4)	62.7 (61.4, 64.1)	62.9 (60.5, 65.3)	60.8 (59.4, 62.1)
HDL-cholesterol, mmol/L	1.3 (1.3, 1.4)	1.3 (1.2, 1.4)	1.3 (1.3, 1.4)	1.4 (1.3, 1.4)
Triglycerides, mmol/L	0.8 (0.8, 0.9)	0.9 (0.8, 1.0)	0.8 (0.7, 0.9)	0.7 (0.6, 0.8)
Insulin resistance, HOMA score	2.1 (1.96, 2.2)	2.2 (2.0, 2.4)	2.2 (1.6, 2.9)	1.6 (1.3, 1.9)

Data for continuous variables are shown as mean (95% confidence interval).

Data for categorical variables are shown as prevalence (95% confidence interval).

### Relation between MVPA and cardiometabolic risk factors

Correlation coefficients (r values) between the MVPA and cardiometabolic risk factors were as follows: 0.16 for BMI, 0.16 for waist circumference, 0.11 for systolic blood pressure, 0.13 for diastolic blood pressure, -0.12 for HDL-cholesterol, 0.06 for triglycerides, 0.22 for insulin resistance, and 0.23 for the metabolic syndrome. Thus, 0.4% (triglycerides) to 5.3% (metabolic syndrome) of the variance in the cardiometabolic risk factors was explained by MVPA.

Differences in cardiometabolic risk factor percentile scores across the 3 physical activity groups are shown in Table 2. The data in this table represent the parameter estimates (b) from the regression models, with the *Frequently Active* group acting as the referent. Thus, parameter estimates greater than 0 indicate higher percentile scores than in the *Frequently Active* group; parameter estimates less than 0 indicate lower percentile scores.

**Table 2:** Differences in cardiometabolic risk factor percentile scores across the physical activity groups: 6–19 year old 2007/09 Canadian health measures survey participants

**Cardiometabolic risk factor**	***Insufficiently active*<60 min/day MVPA on average, b (95% CI)**	**≥60 min/day MVPA on average**	
		***Infrequently active*≥60 min on 1–4 days, b (95% CI)**	***Frequently active*≥60 min on 5–7 days, b (95% CI)**
BMI			
Model 1^a^	5.69 (−0.66, 12.05)	−2.59 (−9.63, 4.46)	referent
Model 2^b^	2.66 (−5.30, 10.62)	−3.78 (−12.63, 5.07)	referent
Waist circumference			
Model 1^a^	5.34 (−2.69, 13.36)	−3.93 (−10.08, 2.21)	referent
Model 2^b^	0.65 (−5.20, 6.50)	−5.78 (−12.86, 1.30)	referent
Systolic blood pressure			
Model 1^a^	4.19 (0.51, 7.87)^c^	0.94 (−9.41, 11.29)	referent
Model 2^b^	−3.80 (−12.49, 4.88)	−2.21 (−12.88, 8.46)	referent
Diastolic blood pressure			
Model 1^a^	6.45 (2.42, 10.48)^c^	6.66 (−5.42, 18.75)	referent
Model 2^b^	2.01 (−5.52, 9.54)	4.91 (−7.16, 16.99)	referent
HDL-cholesterol			
Model 1^a^	−6.45 (−12.78, -0.13)^c^	−5.58 (−15.87, 4.72)	referent
Model 2^b^	−3.46 (−14.29, 7.36)	−4.40 (−15.42, 6.62)	referent
Triglycerides			
Model 1^a^	1.04 (−1.10, 3.18)	0.90 (−2.09, 3.89)	referent
Model 2^b^	−0.13 (−3.52, 3.26)	0.44 (−3.07, 3.94)	referent
Insulin resistance			
Model 1^a^	5.79 (−0.54, 12.13)	5.29 (0.15, 11.74)^c^	referent
Model 2^b^	6.52 (−4.14, 17.17)	6.10 (−0.10, 12.30)	referent
Metabolic syndrome			
Model 1^a^	5.95 (0.15, 11.74)^c^	5.03 (−2.49, 12.55)	referent
Model 2^b^	0.65 (−5.27, 6.54)	3.30 (−4.56, 11.16)	referent

Data presented as parameter estimate (b) and the 95% confidence interval for these estimates as obtained from generalized linear models predicting percentile scores of cardiometabolic risk factors from the physical activity groups. The Frequently *Active* group acted as the referent (b = 0.00) for all analyses.

^a^ Model 1 controlled for visible minority status and household income.

^b^ Model 2 controlled for variables in Model 1 + volume of MVPA (average minutes per day).

^c^ Significantly different from referent group (*P* < 0.05).

After controlling for the covariates in Model 1, a less favorable overall cardiometabolic risk factor profile was noted in the *Insufficiently Active* group than in the *Frequently Active* group (Table 2). With the exception of triglycerides, the percentile scores were 4 to 6 units higher (lower for HDL-cholesterol) in the *Insufficiently Active* group. There was a substantial variability for most of the parameter estimates, and the differences between the *Insufficiently Active* and *Frequently Active* groups only reached statistical significance (*p* < 0.05) for 4 of the 8 risk factors.

After controlling for the covariates in Model 1, there were noticeable differences (5 to 7 percentile units) in the average diastolic blood pressure, HDL-cholesterol, insulin resistance, and metabolic syndrome scores between the *Infrequently Active* and *Frequently Active* groups. However, the only statistically significant (*p* < 0.05) difference was for insulin resistance, wherein a 5.29 (95% CI: 0.15, 11.74) higher percentile score was observed in the *Infrequently Active* group.

To explore whether the differences in cardiometabolic risk factors between the *Infrequently Active* and *Frequently Active* groups were attributable solely to the 21 minutes/day higher average MVPA levels in the *Frequently Active* group, or whether there was an independent effect of the daily frequency of being active for ≥60 minutes, additional models were run that controlled for the average minutes/day of MVPA (see Model 2 in Table 2). An attenuation of the percentile scores in model 2 vs. model 1 would indicate that the differences between these two groups in model 1 were attributable to the higher average MVPA levels in the *Frequently Active* group. The general pattern of findings was that the percentile scores in model 2 for the *Infrequently Active* group were not closer, or were only marginally closer (i.e., slightly attenuated), to those of the *Frequently Active* group than they were for model 1.

In an additional model we considered whether the addition of BMI percentile score as an additional covariate influenced the associations observed in Model 2 of Table 2. No discernible differences in the results were observed versus those reported for Model 2 (data not shown).

## Discussion

Our primary objective was to compare cardiometabolic risk factors in physically active children and youth who accumulated their weekly MVPA in different patterns. A key finding was that *Infrequently Active* children and youth – children and youth who accumulated 60 minutes of MVPA on average per day but who met this 60 minute target <5 days per week – were more insulin resistant than *Frequently Active* children and youth. This is an important observation given the strong, independent effects that insulin resistance has on the development of type 2 diabetes [36,37] and coronary heart disease [38,39]. The differences in insulin resistance between *Infrequently Active* and *Frequently Active* children and youth appeared to be attributable to differences in the weekly frequency of meeting the daily target of 60 minutes of MVPA rather than to differences in the weekly volume of MVPA. There were also notable differences in diastolic blood pressure, HDL-cholesterol, and metabolic syndrome scores between the *Infrequently Active* and *Frequently Active* children and youth, although these differences were not statistically significant.

To our knowledge, no previous pediatric studies have investigated the independent health influence of the weekly fractionalization of MVPA. Previous population-based studies of children and youth from the United States and Europe have documented a relationship between average daily MVPA, as measured by accelerometer, with individual cardiometabolic risk factors [29,40] and summary cardiometabolic risk factors scores [31,41]. Our findings that physically active Canadian children and youth tend to have a more favorable overall cardiometabolic risk factor profiles than their insufficiently active counterparts is consistent with these prior observations. These findings also support the new physical activity guidelines of the World Health Organization [1], United States [2,3], and Canada [4] which recommend that school-aged children and youth accumulate ≥60 minutes of MVPA on a daily basis. A unique recommendation within Canada’s guidelines is that “More physical activity provides greater health benefits” [4]. In other words, while children and youth should strive to accumulate 60 minutes daily, those who accumulate >60 minutes will achieve even greater health benefits. This was also supported by findings of our study. In particular, the average insulin resistance percentile score of *Infrequently Active* children and youth, who had an average daily MVPA of 70 minutes, was 5.29 units higher than in *Frequently Active* children and youth, who had an average daily MVPA of 91 minutes (see Table 2).

It has been well documented that MVPA has acute effects on several cardiometabolic risk factors [11]. The acute effects on lipids and lipoproteins are most pronounced for triglycerides and HDL-cholesterol. These effects occur 18–24 hours after exercise and persist for up to 72 hours [11]. Reductions in systolic and diastolic blood pressure occur immediately after exercise; however, these changes are short-lived and last for 16 hours or less [11]. The acute effects on insulin resistance are also immediate, but can last for 3 or 4 days [11]. Based on these observations, it was not surprising that the differences in insulin resistance between the *Infrequently Active* and *Frequently Active* groups remained after adjusting for differences in the volume of MVPA across these two groups.

Our findings have potential public health implications. Namely, they suggest that, as implied in current physical activity guidelines, engagement in 60 minutes of MVPA on a daily or near daily basis is beneficial to the cardiometabolic health of children and youth. In other words, from a health perspective, it appears to be more beneficial for a young person to accumulate 7 hours of weekly MVPA over 7 days than over 4 days. Furthermore, encouraging children and youth to be active on a daily basis may help them accumulate more activity over the course of a week. In this study *Frequently Active* participants accumulated 146 more minutes of MVPA over the week than *Infrequently Active* participants.

This study had several limitations. First, the cardiometabolic risk factors were assessed in the clinic visit on the day prior to the 7-day MVPA measurement period. Therefore, when assigning participants to MVPA groups, we assumed that the MVPA patterns were consistent in the week prior to and following the clinic visit. Second, for participants with less than 7 days of valid accelerometer wear time we made assumptions about their weekly pattern of physical activity when assigning them to a physical activity group. It is unclear if this led to differential or non-differential misclassification of the exposure variable, and therefore whether the observed associations were under or overestimated. Third, this was a cross-sectional study that does not provide temporal evidence. Fourth, a significant percentage of the CHMS sample was excluded from the analyses because of incomplete or missing data. This decreased the sample size and we may have been underpowered for some of the analyses, particularly those that compared the *Infrequently Active* (N = 108) and *Frequently Active* (N = 184) groups. Firth, because of sample size limitations, we could not perform the analyses separately by sex or age group. Finally, the accelerometers used in the CHMS do not accurately capture physical activities that are not step-based such as swimming, cycling, and weight training [42].

## Conclusion

Our findings support the notion that the fractionalization of MVPA throughout the week is important for cardiometabolic health, at least for insulin resistance. These findings set the stage for tightly controlled intervention studies, which could more adequately address the relevance of the weekly fractionalization of MVPA.

## Abbreviations

CI: Confidence interval; cpm: Counts per minute; BMI: Body mass index; HDL: High density lipoprotein; HOMA: Homoeostasis model assessment; MVPA: Moderate-to-vigorous physical activity.

## Competing interests

The authors declare that they have no competing interests.

## Authors’ contributions

IJ led the design the study, secured funding, provided advice on the statistical analyses, and wrote the first draft of the paper. SW helped design the study, conducted the statistical analyses, and provided critical feedback on the drafts of the paper. MT and RC helped design the study and provided critical feedback on the drafts of the paper. All authors read and approved the final manuscript.

## Pre-publication history

The pre-publication history for this paper can be accessed here:

http://www.biomedcentral.com/1471-2458/13/554/prepub
